# Influence of Landscape Diversity and Composition on the Parasitism of Cotton Bollworm Eggs in Maize

**DOI:** 10.1371/journal.pone.0149476

**Published:** 2016-02-16

**Authors:** Bing Liu, Long Yang, Yizhong Yang, Yanhui Lu

**Affiliations:** 1 College of Horticulture and Plant Protection, Yangzhou University, Yangzhou, China; 2 State Key Laboratory for Biology of Plant Diseases and Insect Pests, Institute of Plant Protection, Chinese Academy of Agricultural Sciences, Beijing, China; Institute of Plant Physiology and Ecology, CHINA

## Abstract

We deployed >50,000 *Helicoverpa armigera* eggs in maize fields to assess the rate of parasitism by *Trichogramma chilonis* across 33 sites during a three-year span (2012–2014) in northern China. Subsequently, we used a partial least squares (PLS) regression approach to assess the relationship of landscape diversity with composition and parasitism potential. The parasitism rate of *H*. *armigera* eggs by *T*. *chilonis* ranged from 0–25.8%, with a mean value of 5.6%. Landscape diversity greatly enhanced parasitism at all four different spatial scales (0.5, 1.0, 1.5 and 2.0 km radius). Both the proportion of arable area and the total planting area of two major crops (cotton and maize) had a negative correlation to the parasitism rate at each scale, whereas parasitism was positively correlated to the proportion of host crops of *H*. *armigera* other than cotton and maize at the 0.5 to 2.0 km radius scales as well as to that of non-crop habitat at the 0.5 and 1.0 km radius scales. The study indicated that maintaining landscape diversity provided an important biocontrol service by limiting *H*. *armigera* through the egg parasitoid *T*. *chilonis*, whereas rapid agricultural intensification would greatly reduce the presence and parasitism of *T*. *chilonis* in China.

## Introduction

Agricultural sustainability depends largely on the ability of natural enemies to suppress pest populations [1]. The biocontrol services provided by natural enemies can not only control crop pests [2] but also reduce the need for chemical pesticides in cropping practices [3, 4], and decrease the potential for pesticide residue in plant-derived foods and the environment, slow the development of pest resistance to pesticides, and control secondary pest resurgence [5]. However, this important ecosystem services has received limited attention from researchers and the public in many parts of developing countries, including China [6].

Previous studies indicate that biocontrol services are greatly affected by landscape contexts, especially by landscape composition [7–9]. Heterogeneous habitats possess diverse ecosystem functions [10]. Among these functions, primary crops usually provide the major insect source of prey and act as hosts for natural enemies [11], whereas non-crop habitats or perennial habitats often support floral nectars and alternative prey species, and provide temporary refuges and overwintering sites [12, 13]. Biocontrol services also vary at multiple spatial scales [14–17], which are usually related to the dispersal capability of various natural enemies [18]. Generally, predators such as ladybeetles fly longer distances than parasitoid wasps [19, 20]; thus, parasitism has stronger spatial scale effects than does predation [21]. Recently, rapid agriculture intensification has led to an obvious simplification of agricultural landscapes all over the world, resulting in a marked reduction in the sustainability, stability and diversity of ecosystems [22–24] while simultaneously increasing the instability of relationships among food webs of crops, pests and natural enemies and their associated biocontrol services [25, 26]. For example, increasing the amount of corn planted for biofuel production reduced ecosystem services of natural enemies for controlling *Aphis glycines* in soybean fields in the United States [2]. In China, the cropping structures and ecological conditions in agri-ecosystems are changing rapidly; however, the effect of this agricultural intensification on biocontrol services has not been widely assessed at landscape levels in China [27, 28].

During the early 1990s, the cotton bollworm *Helicoverpa armigera* was the most serious insect pest on cotton, maize, peanut, soybean, vegetables and many other crops in China [29, 30]. After the widespread commercial application of transgenic cotton expressing Bt (*Bacillus thuringiensis*) toxin, the bollworm population density in cotton and other neighboring crops dramatically decreased [29]. However, *H*. *armigera* remains a common pest on non-Bt crops in China, including maize, peanuts, and vegetables. In this study, we estimated the effect of landscape context on the parasitism of *H*. *armigera* eggs in maize fields to address the following three questions. (1) Does landscape diversity and composition affect parasitism? (2) Do biocontrol services have spatial scale effects? (3) How will biocontrol services change as agriculture intensifies? We found that high landscape diversity and high proportion of host plants other than main crops (cotton and maize) greatly enhanced the parasitism of *H*. *armigera* eggs.

## Materials and Methods

### Ethics Statement

This field trial was approved by the local farmers. No specific permits were required for the described field studies, and our study did not involve endangered or protected species.

### Study sites

The study was conducted at a total of 33 field sites distributed around the city of Langfang in Hebei Province and around the cities of Wuqing and Jinghai in Tianjin Province of northern China (11 sites in 2012, 6 sites in 2013 and 16 sites in 2014). All selected sites were planted with maize along a gradient of proportion between pure maize and landscape diversity. In each year of the study, a minimum distance of 4 km separated the selected field sites to prevent any disturbance to the studied maize fields from affecting adjacent sites. We also avoided overlapping sites during the study. To simplify parasitism assessment, the field experiments were conducted from middle-to-late August, when the 4^th^ generation of *H*. *armigera* emerged, during each of the three years (2012–2014). We ensured that no insecticide was sprayed during the trials, and we reimbursed farmers for yield losses at all sites.

### Landscape analysis

The geographical coordinates of each focal maize field site were obtained using a handheld GIS product (Model “MG768”, Beijing UniStrong Science & Technology Co., Ltd., China). We defined our study regions at radii of 0.5–2.0 km with a 0.5 km interval around each site by using open-access satellite imagery from Google Earth. The land-cover map within the 2.0 km radius was digitized using ArcGIS 10.0 software [31] based on ground verification. We dropped small polygons such as ditches or very small features that were smaller than 5 m^2^ and could not be located during ground verification; the total area of all the unidentified field polygons was less than 0.1% of each landscape.

Land cover was assigned to one of 15 categories that were combined into three primary classes for landscape analysis: (1) Arable crops (the dominated crops cotton and maize as well as other host crops such as peanut, soybean, sweet potato, vegetables and fruit trees) and (2) Non-crop habitats (included forests, shrublands and grasslands), and (3) Urban which included buildings, roads, abandoned land, other impervious surfaces, and water. The digitized polygon land cover layers for each site were converted to a grid format in ArcGIS, and then the area and proportion of each selected site were determined with Fragstats 4 software [32] at each spatial scale.

We quantify landscape heterogeneity by using Simpson’s diversity index (*D*) which can effectively describe the variance in the proportion of area covered by different land-cover categories and can be calculated for each spatial scale [14]. Simpson’s diversity, *D*, is calculated by *D* = 1/Σ(*pi*)^2^, where *p*_*i*_ is the proportion of the *i*th land-cover category based on the 15 landscape categories.

### Sentinel insect

We used *H*. *armigera* eggs as the sentinel host for parasitoid *T*. *chilonis*. Adult *H*. *armigera* were collected at night using artificial lights. We established a laboratory colony at the Langfang Experimental Station (Institute of Plant Protection of the Chinese Academy of Agricultural Sciences, Hebei province, China). The moths in the colony were maintained at 25 ± 1°C, 60 ± 10% RH and 16:8 h (L:D). Larvae were reared on an artificial diet, the main components of which were wheat germ, casein and sucrose. Adult moths were fed a 20% honey solution after emergence. In the laboratory, gauze is used to collect eggs because the moths prefer to lay eggs on it [33]. New gauze was placed in an oviposition cage containing female and male adults at 18:00 pm when moths were ready to begin oviposition. The next morning (6:00 am) we collected the gauze with sentinel eggs attached. This “egg gauze” was cut into small pieces (approximately 3 cm wide and 5 cm long), and any flat (unfertilized) eggs on it were removed using an insect pin under a stereomicroscope. Each piece of “egg gauze” contained 50–100 eggs were used in field studies between 8:00–12:00 am on the same day. We randomly deployed 10 pieces of selected “egg gauze” as 10 replications at each study site for each deployment time. Due to low background pest density [29], the number of sentinel eggs was greatly higher than that naturally occurred in focal field, and ensured that it was sufficient to assess parasitism.

### Experimental methods

We selected a large plot that ranged from 3000–10000 m^2^ in the center of each maize field site and then randomly selected a total of 10 maize plants with a minimum 10 m interval between each plant and the field border. For each selected plant, one piece of “egg gauze” was attached to a young leaf near the middle of the maize stalk using an insect pin for each trial. The egg gauze was carefully collected from the field after 48 h of exposure and maintained within 10 ml glass dactylethrae. Then, a new egg gauze was deployed onto the same plant and similarly collected after another 48 h in the field. Three consecutive egg gauze deployments per site were conducted every year. During the summer, the developmental period for *H*. *armigera* eggs is approximately 72 h [30]; thus, after collecting the gauze from the field, we immediately recorded the number of eggs remaining on each piece of gauze prior to egg hatching because some of the eggs might have fallen off during the experimental operation or been damaged by predators. By 24 h after collection, nearly all the unparasitized eggs hatched successfully. By this point, all parasitized eggs darken to black and any remaining dead eggs shrivel due to water loss. At this time, we counted the number of parasitized eggs present per egg gauze. The collected “egg gauzes” were kept at 25 ± 1°C, 60 ± 10% RH and 16:8 h (L:D) and observed once per day. As parasitoids emerged, they were collected and placed into a 1.5 ml centrifuge tube containing 95% alcohol. The parasitoid samples were stored at -20°C for morphological species identification.

### Statistical analysis

We used a partial least squares (PLS) regression approach to model variation between landscape variables and parasitism [34, 35]. The PLS model can minimize sample response prediction error and works by extracting successive linear combinations of the predictors to explain both predictor and response variation, especially when the predictors are highly correlated [36]. The PLS model chooses the number of extracted factors by calculating cross validation (*Q*_*h*_^*2*^). In general, when *Q*_*h*_^*2*^≥0.0975, adding a factor to the model will be significant [37]; however, extracting too many factors can cause overfitting; in other words, tailoring the model too much will be detrimental to future predictions. Here, we used a one-at-a-time validation method to select the number of extracted factors by cross validation; the number of factors that minimize the predicted residual sum of squares (*PRESS*) was considered the best.

After selecting the adapted factors, we assessed the validity of the model by examining the amount of variation in the matrix of predictor and response variables using three indices (*R*^*2*^_*X*_, *R*^*2*^_*Y*_ *and Q*^*2*^). *R*^*2*^_*X*_ is the proportion of variance in the matrix of predictor variables used in the model, *R*^*2*^_*Y*_ is the proportion of variance in the response variable explained by the model, and *Q*^*2*^ is the proportion of variance in the response variable that can be predicted by the model.

Next, the relative influence of each predictor variable was estimated using the Variable Importance for Projection (*VIP*), which is the sum of the influence of the variable over all the model dimensions divided by the total variation explained by the model. The *VIP* represents the contribution of each predictor in fitting the PLS model for both predictors and responses. In fact, variables are significantly explained when *VIP* ≥0.8 [36, 38]. Because predictor and response variables were standardized, the coefficients (*B*) of this equation may be interpreted as the influence of a particular predictor variable on the response variable, providing a direct indication of which predictors are most useful for predicting the dependent variable. If a predictor has a relatively small coefficient (|*B|*) and *VIP* (<0.8), it is a prime candidate for removal [38], after which the PLS procedures should be re-run to obtain a better model. The correlations loading plot shows how much variation in each variable is accounted for by the first two factors, jointly by the distance of the corresponding point from the origin and individually by the distance for the projections of this point onto the horizontal and vertical axes [39].

Before implementing this PLS regression (PROC PLS), we first tested the normality of the data and determined whether the variables were symmetrical. When they are not, an arcsine square root transformation of the predictors (percentage data) and response variable (parasitism rate) should be conducted to achieve data standardization. Ultimately, eight predictor variables (Simpson, Cotton, Maize, CM, OHC, Arable, NCH, Urban; see variable description in Table 1) were used in the model. We calculated the mean value of the parasitism rate on *H*. *armigera* eggs per site for each of the three deployment times, which served as response variables in the analysis. The PLS analysis was performed using SAS 9.3 software [39].

**Table 1:** The landscape variables at each spatial scale used in Partial Least Squares (PLS) regression analysis.

Predictor name (abbreviation)	Description
Landscape diversity index (Simpson's)	Simpson's diversity index, which indicates landscape heterogeneity.
Cotton	The percentage land cover of the main crop of *H*. *armigera*
Maize	The percentage land cover of another main crop of *H*. *armigera*
Cotton and maize (CM)	The percentage land cover of the main crops of *H*. *armigera*, including cotton and maize
Other host crops (OHC)	The percentage land cover of the other host crops of *H*. *armigera* such as peanut, soybean, sweet potato, vegetables and fruit trees
Arable	The percentage land cover of all arable host crops, including the main crops and other host crops
Non crop habitat (NCH)	The percentage land cover of non-crop habitat such as forest, shrublands and grasslands
Non vegetation land (Urban)	The percentage land cover of buildings, roads, abandoned land, other impervious surface, and water

## Results

### Parasitism on sentinel eggs in maize fields

Over the three years of the study, we deployed more than 50,000 sentinel eggs to investigate parasitism in the center of maize fields in all 33 study sites. A total of 2,000 wasps of *T*. *chilonis* emerged from the parasitized sentinel eggs. The parasitism rate of sentinel eggs varied from 0–25.8%, with a mean value of 5.6% at each site.

### Landscape composition

The term “landscape” in this study represented a gradient of agriculturally dominated land; the landscape varied at different spatial scales. Arable crop was the primary landscape type across all scales, varying from 27.9–96.9%. Maize and cotton were the main host crops for *H*. *armigera* with a range from 20.7–96.9%; other host crops were less prevalent, ranging from 0–27.6%. Moreover, the percent of non-crop habitat ranged from 0.4–57%. Urban land is an abiotic variable in the landscape system, with a range of 0.7–61.2%. We used Simpson’s diversity as the indication of landscape composition, the range of the Simpson value was 1.27–3.15 (Table 2).

**Table 2:** Landscape diversity index (Simpson’s *D*) and the proportion of other landscape variables at four spatial scales across all 33 sites.

Radius (km)	Landscape variables	Simpson's *D* value and land proportion at each scale (%)		
		Minimum	Maximum	Average
0.5	Simpson’s	1.27	4.61	2.59
	Cotton	1.97	88.44	24.90
	Maize	7.14	82.80	45.52
	CM	20.71	96.85	70.42
	Arable	27.89	96.85	74.58
	OHC	0.00	27.58	3.81
	NCH	0.41	56.38	11.95
	Urban	0.67	61.29	13.47
1.0	Simpson’s	1.53	5.71	2.98
	Cotton	0.92	68.84	17.74
	Maize	4.75	80.47	46.36
	CM	31.70	92.82	64.10
	Arable	42.94	93.31	68.44
	OHC	0.00	16.90	3.92
	NCH	2.25	54.68	12.93
	Urban	2.38	53.93	18.64
1.5	Simpson’s	1.73	7.29	3.14
	Cotton	0.41	68.91	16.67
	Maize	9.59	74.75	45.27
	CM	33.02	82.30	61.94
	Arable	36.81	83.48	65.97
	OHC	0.00	15.79	3.54
	NCH	1.95	56.28	12.49
	Urban	6.66	50.44	21.54
2.0	Simpson’s	1.87	6.74	3.15
	Cotton	0.23	62.16	13.92
	Maize	7.87	71.99	45.63
	CM	25.69	79.31	59.56
	Arable	30.94	79.31	63.39
	OHC	0.00	17.97	3.27
	NCH	2.79	56.98	12.34
	Urban	8.84	48.74	24.28

### Influence of landscape composition on parasitism services

The first two-factor model explained the cumulative variations of 65.2%, 59.9%, 54.9% and 55.5% in landscape predictor variables as well as the 35.6%, 25.7%, 40.7% and 42.8% variations in parasitism rate at the 0.5, 1.0, 1.5 and 2.0 km radius scales, respectively. Factor 1 explained 40.7%, 42.6%, 44.4% and 44.7% of the variation in landscape predictor variables (R^2^_X_) and 27.5%, 21.1%, 27.8% and 29.0% of the variation in parasitism rate (R^2^_Y_), whereas Factor 2 explained 24.5%, 17.3%, 10.5% and 10.8% of the variation in landscape predictor variables (R^2^_X_) and 8.1%, 4.6%, 12.9% and 13.8% of variation in parasitism rate (R^2^_Y_) at the four spatial scales, respectively (Table 3).

**Table 3:** Percent of variation accounted for by Partial Least Squares (PLS) regression analysis of the parasitism rate.

Radius (km)	Model Variables	R_1_^2^	R_2_^2^	Cumulative
0.5	R^2^_X_	40.7	24.5	65.2
	R^2^_Y_	27.5	8.1	35.6
1.0	R^2^_X_	42.6	17.3	59.9
	R^2^_Y_	21.1	4.6	25.7
1.5	R^2^_X_	44.4	10.5	54.9
	R^2^_Y_	27.8	12.9	40.7
2.0	R^2^_X_	44.7	10.8	55.5
	R^2^_Y_	29.0	13.8	42.8

Note: The percentage of variation in each predictor variable explained by Factors 1, 2, and the cumulative variation explained are provided in the R_1_^2^, R_2_^2^ and cumulative columns, respectively. R^2^_X_ and R^2^_Y_ provide the cumulative amount of variation explained for all predictor and response variables.

**Fig 1:**
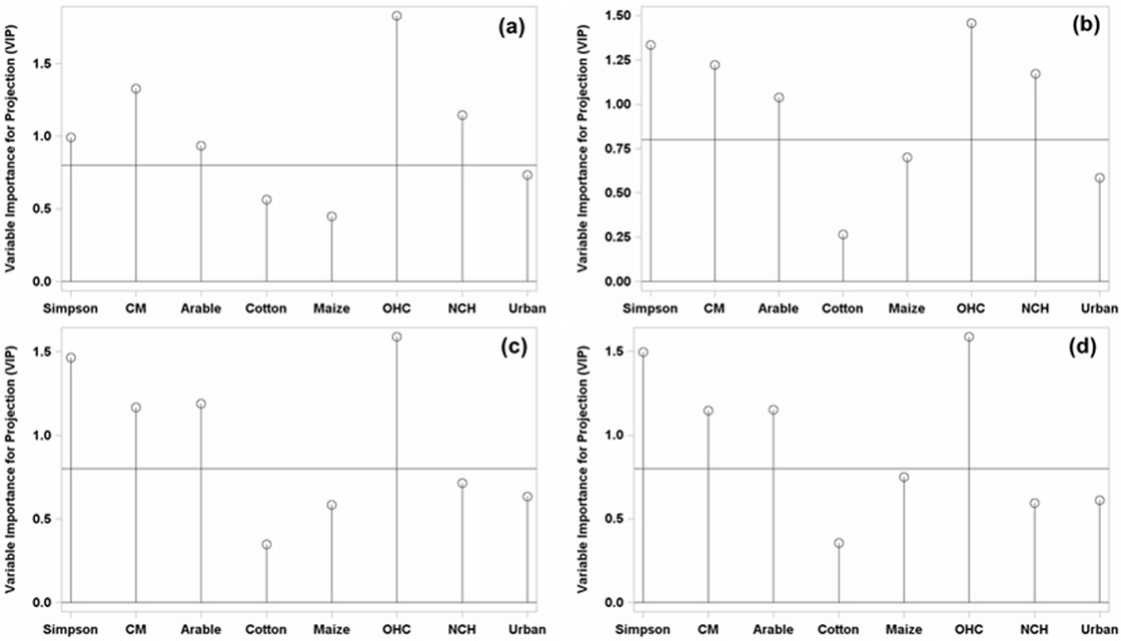
Needle plots describe the Variable Importance for Projection (*VIP*) value of different landscape variables at four spatial scales for the response variable (parasitism rate). Radius (a): 0.5 km, (b): 1.0 km, (c): 1.5 km, (d): 2.0 km. The line parallel to the X axis indicates Wold’s Criterion (*VIP* = 0.8).

**Fig 2:**
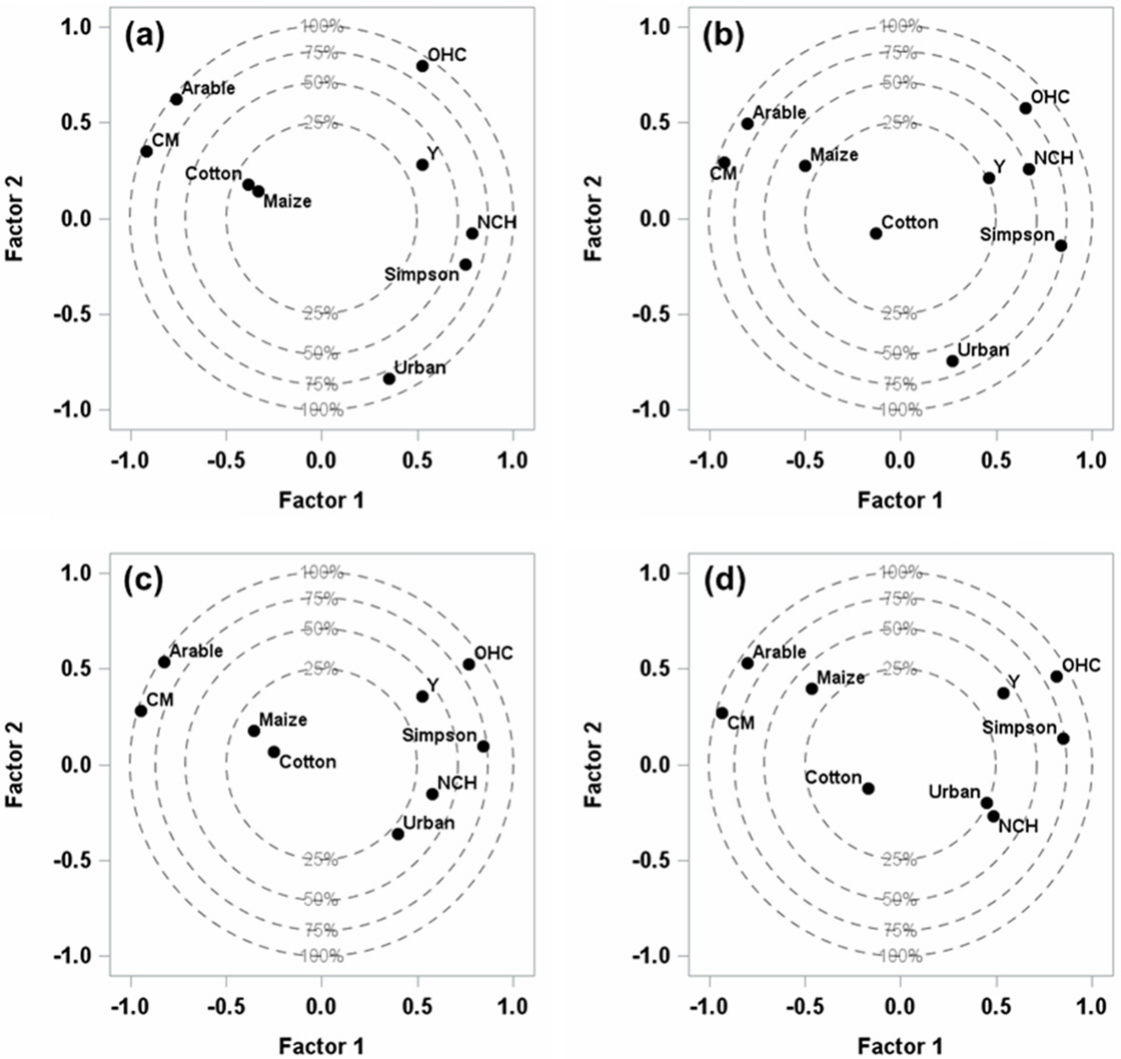
Correlation loadings for the plot of the response variable (parasitism rate, Y) and predictor variables across four spatial scales based on PLS analysis. Radius: (a) 0.5 km; (b) 1.0 km; (c) 1.5 km; (d) 2.0 km. The four dotted line circles (25%, 50%, 75% and 100%) indicate the percentage range of variation explained by the first two factors. The distance of marked points from the origin and from the horizontal and vertical axes jointly show the amount of variation in each variable accounted for by the first two factors.

Furthermore, the four common landscape variables had a significant *VIP* score ≥0.8 at all four radius scales (Fig 1a and 1d), in which the parasitism rate was positively related to two variables: host crops of *H*. *armigera* other than the two major ones (cotton and maize) (OHC) and the landscape diversity index (Simpson). In contrast, the parasitism rate was negatively related to the total area percentage of cotton and maize (CM) as well as to the arable area (Fig 2a and 2d). The coefficients (*B*) of the PLS model also described the importance each predictor had in predicting the response (Table 4). The other three landscape variables (Urban, Cotton and Maize) had no significant correlation to the parasitism rate; they had *VIP* scores of <0.8 (Fig 1a and 1d) and so had low coefficients (*B*, Table 4) across all four radius scales. Another landscape variable, non-crop habitat (NCH), had high *VIP* values (*VIP* ≥0.8, Fig 1a and 1b) and was also positively related to the parasitism rate at the 0.5 and 1.0 km scales (Fig 2a and 2b) but had small *VIP* values (*VIP* <0.8, Fig 1c and 1d) and no significant correlation to the response variable at the 1.5 and 2.0 km radius scales (Fig 2c and 2d).

**Table 4:** Model output for predicted (landscape) and response (parasitism rate) variables used in the Partial Least Squares (PLS) regression analysis.

Radius (km)	Variables	*B*	R_1_^2^	R_2_^2^	Cumulative
0.5	**Simpson’s**	**0.0884**	**55.9**	**5.8**	**61.7**
	**CM**	**-0.1602**	**84.3**	**12.5**	**96.8**
	**Arable**	**-0.0083**	**57.9**	**38.9**	**96.8**
	Cotton	-0.0699	14.9	3.2	18.0
	Maize	-0.0432	11.1	2.0	13.1
	**OHC**	**0.3716**	**27.8**	**63.5**	**91.3**
	**NCH**	**0.1417**	**61.5**	**0.6**	**62.1**
	Urban	-0.1211	12.5	69.5	82.0
1.0	**Simpson’s**	**0.1754**	**69.8**	**1.9**	**71.8**
	**CM**	**-0.0801**	**85.2**	**8.7**	**93.9**
	**Arable**	**0.0016**	**63.9**	**24.7**	**88.6**
	Cotton	-0.0430	1.7	0.6	2.3
	Maize	0.0390	24.9	7.5	32.4
	**OHC**	**0.2470**	**42.8**	**33.5**	**76.3**
	**NCH**	**0.1712**	**44.9**	**6.6**	**51.5**
	Urban	-0.0818	7.4	55.0	62.5
1.5	**Simpson’s**	**0.3549**	**71.3**	**0.9**	**72.3**
	**CM**	**-0.0427**	**89.4**	**8.0**	**97.4**
	**Arable**	**0.1377**	**67.9**	**28.6**	**96.4**
	Cotton	-0.0622	6.2	0.4	6.6
	Maize	0.0943	12.7	3.2	15.9
	**OHC**	**0.4241**	**59.2**	**27.6**	**86.8**
	NCH	0.0540	33.1	2.3	35.4
	Urban	-0.0985	15.6	13.1	28.7
2.0	**Simpson’s**	**0.3731**	**71.9**	**1.9**	**73.9**
	**CM**	**-0.0547**	**87.8**	**7.3**	**95.1**
	**Arable**	**0.1400**	**64.0**	**27.9**	**91.9**
	Cotton	-0.0963	3.0	1.5	4.5
	Maize	0.1237	21.4	15.7	37.1
	**OHC**	**0.4200**	**65.8**	**21.0**	**86.8**
	NCH	0.0157	23.5	7.3	30.8
	Urban	-0.0564	20.1	4.0	24.1

Note: The percentage of variation in each predictor variable explained by Factors 1, 2, and the cumulative variation explained are listed in the R_1_^2^, R_2_^2^ and cumulative columns, respectively. The *B* values indicate the coefficient of the model; bold text indicates a *VIP* ≥0.8.

## Discussion

Although *T*. *chilonis* has been recorded as the main parasitoid on *H*. *armigera* eggs in northern China, limited data are available concerning its control efficiency under field conditions [40]. In this study, parasitism was assessed at 33 sites in northern China, with the highest parasitism rate of 25.8% occurring at a mean value of 5.6% at each site. These results are similar to those obtained by a recent parasitism survey in cotton fields in the same region with the parasitism rate ranging from 0 to 38.8% and at a mean value of 2.5% [41]. It showed that this parasitoid can provide an important suppressive effect on *H*. *armigera*.

Our data confirmed that landscape diversity can significantly improve the parasitism rate on *H*. *armigera* by *T*. *chilonis* in maize fields. The parasitism rate for the sentinel eggs was significantly positively related to the landscape diversity index (Simpson’s D) at four spatial scales with radii of 0.5, 1.0, 1.5 and 2.0 km. Many previous studies have demonstrated that landscape diversity (complexity) has a positive correlation to parasitoid wasp abundance and their biocontrol services. The results here are also similar to those of our previous study, which indicate that highly heterogeneous landscapes can effectively enhance the rates of parasitism on *H*. *armigera* eggs in cotton fields [41]. However, the previous study confirmed high proportion of urban and water could enhanced parasitism[41], but in this study we found other host crops and non-crop habitats can improve it. Roschewitz et al. [42] and Plećaš et al. [15] also found that in contrast to simple landscapes, complex landscapes with higher diversity enhanced the abundance of parasitoids and parasitism on cereal aphids. In total, diverse habitats in the agricultural landscape always provide varied food sources and refuges for parasitoid wasps, thus favoring their population increase and improving their biocontrol services.

In this study, parasitism was not significantly correlated with the proportion of cotton or maize but was negatively correlated with the total proportion of both major crops as well as with arable land at all four radius scales. Another meaningful finding was that the presence of small host crops for *H*. *armigera* other than cotton and maize significantly improved parasitism rates at the four spatial scales tested. *H*. *armigera* is a polyphagous insect with more than 200 host plants in China [30]. In multiple-cropping systems, the various crops have diverse planting seasons, growth stages and agricultural practices, but the agricultural practices of major crops (cotton and maize) are relatively consistent. Hence, crops other than cotton and maize are more available to support populations of *H*. *armigera* through all generations and function as an important source habitat for this parasitoid wasp.

Many studies have reported a positive effect of non-crop habitats on parasitoid abundance [24] and on their biocontrol services [43], but the effect depends on the spatial scales [44]. We found that non-crop habitat surrounding a maize field could significantly enhance the parasitism rate by *T*. *chilonis* from 0.5 to 1.0 km scales, but the effect was not significant at larger scales (such as the 1.5 and 2.0 km radii). Non-crop habitat can provide nectar, pollen sources and refuges with limited human disturbance, all of which naturally enhance the occurrence of parasitoids in agro-ecosystems [45]. Along with many crops, weeds and other vegetation in non-crop habitat also function as host plants for *H*. *armigera* [30], the presence of which also helps support the parasitoid population.

As the dispersal ability of parasitoids is relatively weak, their efficiency as biocontrol services usually changes between small and large spatial scales [21]. In the present study, the Variable Importance for Projection (*VIP*) scores of small crops (other host crops, OHC) were higher than the *VIP* scores for non-crop habitat (NCH) at all four spatial scales (Fig 1a and 1d). Parasitism was correlated to the proportion of small crops at 0.5 to 2.0 km radius scales and to that of non-crop habitat at 0.5 and 1.0 km scales. This finding indicated that small crops other than cotton and maize played a more important role in promoting the population suppression of *H*. *armigera* by *T*. *chilonis*. In general, the proportion of other host crops is larger than the proportion of non-crop habitat nearby the main crop field. The poplar, the dominant tree species in non-crop habitat, is not a host plant for *H*. *armigera* and cannot provide ample nectar and pollen sources. Hence, the ecological effect for conservation biological control of non-crop habitat is less than that of small crops.

Based on the above findings, as agriculture has intensified, landscape diversity has significantly decreased. In particular, a reduction in planting of small crops impairs the ecosystem services function of natural enemies (e.g., the parasitism of *H*. *armigera* by *T*. *chilonis*). Our study provides significant knowledge on pest management practices that utilize ecosystem services of natural enemies to suppress pests and reduce insecticide spraying in China.
